# Stress in action wearables database: A database of noninvasive wearable monitors with systematic technical, reliability, validity, and usability information

**DOI:** 10.3758/s13428-025-02685-4

**Published:** 2025-05-13

**Authors:** Myrte Schoenmakers, Melisa Saygin, Magdalena Sikora, Thomas Vaessen, Matthijs Noordzij, Eco de Geus

**Affiliations:** 1 Department of Biological Psychology, VU Amsterdam, Van Der Boechorststraat 7, 1081 BT Amsterdam, Netherlands; 2 Amsterdam Public Health Research Institute, Amsterdam UMC, Amsterdam, The Netherlands; 3 Department of Psychology, Health and Technology, University of Twente, Enschede, The Netherlands; 4 Center for Contextual Psychiatry, Department of Neurosciences, KU Leuven, Louvain, Belgium

**Keywords:** Wearable, Database, Physiology, Ambulatory, Validation, Reliability, Usability

## Abstract

Ambulatory wearable monitoring of human physiology is increasingly utilized in the fields of psychology, movement sciences, and medicine. With the rapid growth of available consumer- and research-oriented wearables, researchers are faced with a multitude of devices to choose from. It is unfeasible timewise for researchers to determine all relevant technical specifications, available signals, signal sampling details, and (raw) data availability, *and* conduct a search of studies regarding the reliability, validity, and usability of wearables. Thus, selection of wearables for a given study proves highly challenging and will often be unsystematic and uninformed. The 10-year research program Stress in Action initiated a publicly accessible database of wearable ambulatory monitoring devices. We outline the genesis and final structure of the first version of the Stress in Action Wearables Database (SiA-WD) and a summary of the characteristics of the wearables it currently contains. Furthermore, one short-term (2 days) and one long-term (3 months) scenario from the field of stress research are provided with walkthroughs of how the SiA-WD can help select the optimal wearable for a specific research project. Insights gathered include the scarceness of studies testing wearable user-friendliness, inconsistencies in reported validity statistics, and imprecise manufacturer documentation on recorded physiological data such as sampling rate (or window) of signals and parameter extraction. The SiA-WD is the first open-access database to simultaneously include physiological sampling information and technical specifications along with a systematic reliability, validity, and usability search. It will be iteratively expanded to facilitate informed and time-efficient wearable selection. For access to the database, see the following: https://osf.io/umgvp/.

## Introduction

Ambulatory wearable devices are being used in various fields, such as psychology, movement sciences, and medicine, for the continuous monitoring of a multitude of physiological signals in daily life (Pevnick et al., 2018). Ambulatory wearable devices, shortened to wearables hereafter, can be used by (clinical) researchers (Patel et al., 2012, 2021) as well as the public to track physical parameters like activity level, sleep duration, respiration rate, and heart rate variability. These wearables may come in forms such as smart garments with embedded sensors (e.g., Hexoskin by Carré Technologies), smartwatches (e.g., Fitbit Sense 2), rings (e.g., Oura Ring by Oura Health, ResRing by BIOPAC), stick-on electrodes connected to a carry-on central processing unit (VU-AMS 5 fs by Vrije Universiteit Amsterdam), stretchable belts (e.g., Equvital Eq. 02 +), or headbands (Muse 2 by Interaxon) (Majumder et al., 2017; Peake et al., 2018). As the already extensive availability of ambulatory wearables continues to rapidly increase, it becomes challenging to find the optimal wearable for a given research question (Haddad et al., 2020). While validity and reliability are still the most prominent factors to consider in choosing a measurement device, other criteria have considerable pragmatic implications, such as user-friendliness to reduce drop-out rates, data security to avoid data breaches (Areia et al., 2020), and low cost to allow larger-scaled research (Haddad et al., 2020).

In the field of stress research, the ability to monitor continuous physiological outcomes is becoming increasingly important to improve ecological and predictive validity (De Geus & Gevonden, 2024). However, making informed between-device comparisons and selecting the optimal wearable for physiological stress monitoring has proven challenging (Giurgiu et al., 2022; Pevnick et al., 2018). This is due to both the vast number of devices and the time investment associated with examining all reliability, validity, and usability papers per device. Furthermore, the list of devices that meet the required conditions for a study (e.g., specific set of recorded signals) is not always easy to identify through rudimentary Web or literature searches. This may, for example, lead researchers to discover that a device does not allow for raw data extraction only after the purchase of the device or collection of data. Selection of devices is often based on recommendations of colleagues or by relying on the most often used wearables in the existing literature. This leads to the same devices being used time and again, which hampers the adoption of newer technology that may have better signal quality or offer new measures. In the current paper, we consider both physiological signals and parameters. Signals refer to continuous time-series data such as a photoplethysmography or an electrodermal activity sensor recording. Parameters are derived from these signals over particular time windows, and examples include heart rate and skin conductance level.

An overview of wearables in the form of a database systematically compiling the reliability, validity, and usability, amongst other technical information, may assist researchers in choosing the optimal solution to perform continuous measurement of psychophysiological signals in daily life. To date, such a systematic and regularly updated overview is lacking. While there have been attempts to provide an overview of available wearables relevant for a given research field, often in the form of systematic reviews (Iqbal et al., 2016; Lu et al., 2023; Vijayan et al., 2021), these often provided no systematic information on the wearables’ reliability, validity, or usability. They also tend to have a narrow scope on a subset of parameters. For example, in a systematic review of ambulatory monitoring devices for measuring the cardiovascular activity in community-dwelling adults, a list of devices that measured one or more cardiovascular parameters was provided, but important information on the devices’ broader functionality and any other physiological parameters measured was left out (Lu et al., 2023). Furthermore, existing databases are usually not updated iteratively. Just as in printed overviews in journal publications, this static information can become outdated quickly. This problem is particularly salient in view of the ongoing rapid expansion of new (versions of) wearables.

Recognizing these limitations, several attempts have been made to create online databases that would provide a more usable overview of wearable devices for a specific research field or for commercial purposes. In an academic context, the CHIMERA database (Paredes et al., 2021) has been developed to facilitate access to and exchange of information on a wide range of concepts related to wearable technologies. Its main goal was to support a multidisciplinary discourse and collaboration between institutes and companies developing wearables, and to help researchers select the optimal device for their research. However, the database mostly served the development of wearables, not their final use, and is now no longer available online.

As a second example, Henriksen and colleagues compiled a database with 423 consumer-based fitness trackers and watches measuring physical activity. Such a database allows for easier comparison between the devices included because it is possible to filter the data using programming or directly in spreadsheet software. However, information that would be important for daily-life stress research is missing, including information on which stress-related physiological signals are measured, the battery life, availability of raw data, and measurement reliability and validity. Therefore, direct comparison between devices on these aspects is hampered.

A third example of an open access database is the Library of Digital Measurement Products, which has gathered validity and usability evidence of the wearables included. This database provides a dynamic overview of both wearable and ambient (e.g., Wi-Fi sensing) technologies, but the scope of information is still limited. The technical specifications are restricted to *form factor* and *wear location*, with no further details (e.g., available signals) provided. This again makes comparison between devices difficult.

There are also overviews of wearables that are not geared to researchers but are entirely consumer-oriented. These are usually broader in scope and rely predominantly on affiliate marketing models where revenue is generated from readers engaging with their reviews and subsequently making purchases through provided links. These outlets are better at regularly updating their resources and meeting the pace of new developments. Most commonly, such online resources (e.g., https://www.wareable.com/ or https://www.techradar.com/) include reviews of consumer devices, provide an option to compare them using relevant filters, and signal ongoing trends and new incoming wearables. An example of such an online comparison tool is the Vandrico database, which provides information on over 400 devices that can be used for industrial decision support and to facilitate workplace productivity and automatization. However, due to the strong focus on the consumer markets, such websites are not the optimal resource for researchers, as research-oriented ambulatory monitoring devices, as well as more detailed device information relevant to researchers, are commonly not reported. Additionally, they usually concentrate on the technical specifications and the user-based reviews of perceived performance, which cannot be treated as scientific evidence of actual performance.

A gap can be seen between the listings of devices in scientific articles, which easily become outdated, and the up-to-date, online listings, which lack scientific focus. The recently started 10-year research program Stress in Action (stress-in-action.nl) therefore set as one of its goals to create a database of wearable ambulatory monitoring devices, including both consumer- and research-oriented devices, with a comprehensive overview of aspects relevant for research. Online access to the Stress in Action Wearables Database (SiA-WD) will be made available at no cost, and it will facilitate the comparison between different devices through a set of criteria-based filters. The SiA-WD will be continuously updated for the duration of the Stress in Action project.

The primary focus of the SiA-WD is stress research, and the inclusion of the devices is based on the signals related to the physiological stress response. Our goal is to achieve an overview of information comprehensive enough to optimally support stress researchers in selecting the device not only regarding technical aspects, but that best facilitates answering their research question. The SiA-WD aims to support all researchers drawing inference based on autonomic stress reactivity—from diagnostic efforts around biomarkers of psychopathology development (e.g., as central to the work of Beauchaine and Gatzke-Kopp (2012) on cardiac control and impulsivity or emotion regulation) to those focused on efficacy of interventions (e.g., changes in heart rate variability [HRV] as reflective of autonomic regulation and cardiovascular risk in interventions like exercise therapy, in accordance with the neurovisceral integration theory (see for example de Oliveira Matos et al., 2020). Considering the heterogeneous nature of stress research, studies can encompass a wide range of methodological prerequisites and come with different requirements that a device needs to satisfy, including different parameters of interest, which in turn require different measurement techniques. Therefore, the SiA-WD covers a wide range of devices recording physiology at different levels of scientific detail supporting the diversity inherent to stress research. To illustrate the use of the database, we end the paper with two research scenarios (focused on threat-challenge and cross-stressor adaptation hypotheses) showcasing how research questions guide device selection. Moreover, SiA-WD aims to facilitate the selection of the most suitable devices for cohort studies measuring stress in daily life as well as to provide the best candidate devices for subsequent in-depth validation studies. However, the database can also facilitate researchers in selecting wearables for a range of other research topics such as sleep, physical activity, and cardiovascular health. In short, this database will be a scientific resource on available wearable devices for ambulatory assessment of physiology, and will be open-access, applicable to many different research fields, and periodically updated every 6 months for the coming 10 years.

In this paper, we begin by outlining the methods used to structure and populate the database. We then present the database together with its relevant components and provide two research scenarios demonstrating how researchers can use the database to optimally choose wearables given their research project and resources. We end by discussing the insights gathered while creating the first version of the SiA-WD, as well as outlining the future maintenance of the database.

## Methods

Currently, the SiA Wearables Database is implemented as a Microsoft Excel file that is publicly available at https://osf.io/umgvp/. The excel format ensures easy transfer to other formats. The maximal amount of data, even with foreseen future growth, is considered manageable in this format. The information in the cells of this Excel database was structured in a way that would enable easy automated searching, sorting, and analyzing the database with programs such as Python or R. Most of the time, numerical fields or text field from dropdown menus were used to standardize the input. Rarely, open text fields were used to allow additional clarification. For columns containing multiple components, a standardized format was created with semicolons used to separate the different components. For example, a device with an accelerometer with three axes sampling at 1,000 Hz and positioned on the hip is displayed as “1; 3; 1000; hip”; whereas a cell of a device without an accelerometer will display as “0.”

Below, we first give a description of our approach to select wearables to be included in the database (each *row* of the database contains the information of one device), followed by the type of information on these devices to include in the database (structured into the *columns* of the database), and finally the methods to populate the database with the device-specific information (filling in the *cells)*. Since the reliability, validity, and usability of a wearable are essential aspects for researchers, separate subsections describe our strategies for finding, summarizing, and adding this information on the wearables to the database.

### Wearables included in the SiA Wearables Database

The starting point to select wearables to be included into the database was a list of physiological signals and the parameters derived from these signals that have been widely used to study the physiological human stress response (Eckberg, 2003; El-Hamad et al., 2023; Geus & Gevonden, 2024; Geus et al., 1995; Grossman & Svebak, 1987; Henley et al., 2018; Kim et al., 2018; Klimek et al., 2023; Malm et al., 2004; Mukkamala et al., 2015; Neumann & Blanton, 1970; Osei et al., 2024; Rahma et al., 2022; Steptoe et al., 2000; Treadwell et al., 2010; Ward et al., 2012; Wilhelm et al., 2003). Based on the vast body of physiological stress research, we included electrocardiography (ECG), impedance cardiography (ICG), respiration, photoplethysmography (PPG), electrodermal activity (EDA), and blood pressure as our main signals; for more details see Table 1.

**Table 1:** Basic explanation of the signals commonly used in ambulatory stress research

Signal	Details
Electrocardiography (ECG)	ECG records the electrical activity of the heart; i.e., the depolarization and repolarization of the heart muscle. Commonly, a 12-lead ECG is recorded in clinical settings (McStay, 2019), but for ambulatory recordings, a two-lead solution is often used (Krittanawong et al., 2021)
Impedance cardiography (ICG)	ICG records the changes in impedance in the thorax, e.g. caused by respiration and fluctuations in blood volume and flow during contractions of the heart. It can provide information regarding the cardiac and respiratory system (and fluid states) (Parry & McFetridge-Durdle, 2006)
Photoplethysmography (PPG)	PPG signals are based on absorption and/or reflection of light (green, red, or infrared), commonly measured at the wrist (e.g., smartwatches) or finger (e.g., pulse oximeter). The signal can be used to derive cardiac measures such as the heart rate and—when using multiple wavelengths—oxygen saturation (Alian & Shelley, 2014)
Electrodermal activity (EDA)	EDA measures the conductance of the skin using two electrodes. EDA captures the activity of the eccrine sweat glands and is considered to be a pure measure of the sympathetic nervous system (Boucsein et al., 2012)

From these signals, sometimes by combining them, physiological parameters can be extracted and reported by various wearables. Several parameters typically used in physiological stress research are heart rate (HR) (Steptoe et al., 2000) and other parameters measuring cardiac performance like the pre-ejection period, stroke volume, and cardiac output from the combined ECG and ICG (El-Hamad et al., 2023; Henley et al., 2018; Malm et al., 2004; Treadwell et al., 2010), respiration rate and tidal volume from respiratory stretch belts, thoracic impedance, or inductance plethysmography (de Geus et al., 1995; Pattyn et al., 2010; Wilhelm et al., 2003), skin conductance level (SCL) and the frequency of nonspecific skin conductance responses (ns.SCR) from the EDA (Klimek et al., 2023; Neumann & Blanton, 1970; Rahma et al., 2022), and a number of heart rate variability measures like the standard deviation of heart period intervals (SDNN), the root mean square of successive differences (RMSSD), or high-frequency spectral power (HF-HRV) from the ECG or PPG signals (Kim et al., 2018; Osei et al., 2024) sometimes derived in combination with respiration signals to obtain peak–valley respiratory sinus arrhythmia (pv-RSA) (Eckberg, 2003; Grossman & Svebak, 1987). Lastly, measures like systolic blood pressure (SBP), mean arterial pressure (MAP), and diastolic blood pressure (DBP) are obtained through oscillometer cuff-based methods or by using estimation through pulse transit time assessment (Mukkamala et al., 2015; Vrijkotte et al., 2000; Ward et al., 2012).

Apart from these physiological measures, it has been recommended to additionally measure posture, physical activity, and ambient noise level and temperature, as they can confound the ambulatory assessment of many of the above physiological signals and parameters (De Geus & Gevonden, 2024), but can also by themselves reveal effects of stress on behavioral activation (Giakoumis et al., 2012; O’Brien et al., 2017; Sano & Picard, 2013). Therefore, when a device co-records the accelerometer and/or gyroscope signals and skin or ambient temperature, this information is also included in the SiA-WD. Wearable solutions also exist for a number of other signals and parameters such as sleep duration and quality (Irwin, 2023; Romeijn et al., 2012; Sadeghi et al., 2019), ambient light (Akinwande & Kireev, 2019), electrooculography (Moon et al., 2023), electromyography (Ngo et al., 2022), or core body temperature sensing (Dolson et al., 2022). To keep the SiA-WD concise, we did not include wearables that *only* measured these other signals but included wearables that co-record them *in addition to* the physiological parameter(s) of interest in stress research.

Currently, there are hundreds of wearable devices that measure the physiological signals listed above. A pragmatic approach was used to select a subset of these devices for the first iteration of the SiA-WD database. Based on domain knowledge of the authors, we selected a set of 10 well-known wearables already used in research: the Empatica E4, Empatica EmbracePlus, VU-AMS 5 fs, Oura Ring gen3, Hexoskin Proshirt, Mindware Mobile, Novacor Diasys 3 plus, Apple Watch Series 6, Garmin vivosmart 5, and Fitbit charge 5. Adding these first 10 devices to the database bootstrapped the iterative process of selecting the relevant device information to be included in the database, described in more detail below. Subsequently, we used a systematic approach to select both often-used and newer devices through keyword-based searches, which entailed the following:

1. Conducting database searches at PubMed, IEEE Xplore, Web of Science, Scopus, and APA PsycInfo (through Ebscohost) and ACM Digital Library, with the sorting set to “Most recent” while using a search string containing keywords on wearable monitors, stress, and physiological signals. The physiological keywords included those on both signal types and parameters that can be derived using these signals. The search string was as follows: (photoplethysmography OR electrocardiography OR “heart rate” OR “electrodermal activity” OR “skin conductance” OR ns.SCR OR “blood pressure” OR electromyography OR “skin temperature” OR “stress level” OR “stress detection”) AND (wearable).
2. Examining website articles on newly released wearables via *dcrainmaker.com*, *wareable.com*, *techradar.com*, *vandrico.com*, and *wired.com/tag/wearables.*
3. Consulting colleagues and other researchers who work in the field and asking them for nominating wearables, especially newer ones.

The list of wearables obtained in this way was filtered by the inclusion and exclusion criteria listed in Table 2.

**Table 2:** Eligibility criteria for wearable device inclusion in SiA-WD

Inclusion criteria
• The device is available on the market or was recently discontinued but is still widely used in research • The device measures physiological parameters relevant to stress research • The device is wearable, mobile, and can be used in daily life • Specified information on the device and its components is available in English, either on the manufacturer’s website or in the form of a digital manual
Exclusion criteria
• The device is in development and prototype phase and not readily available • The device has a singular focus on measuring gross body movement • The device is predominantly intended for medical use

**Fig. 1:**
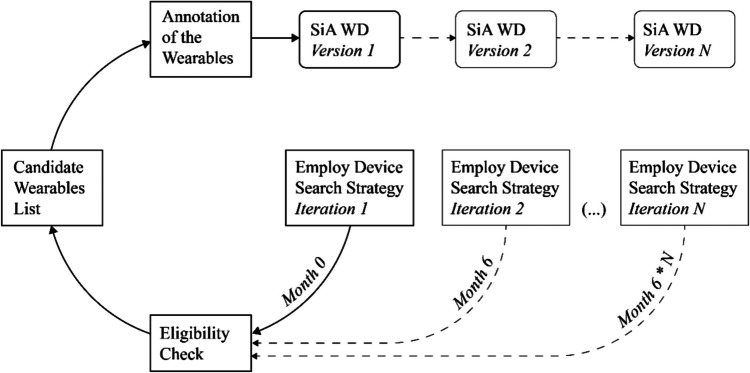
Iterative process of populating the SiA Wearables Database

This process led to the first 172 candidate wearables listed in the database at https://osf.io/umgvp/. As illustrated in Fig. 1, the above procedure will be replicated every 6 months for at least the duration of the Stress in Action project, resulting in updated versions of candidates for the SiA-WD. Using a majority voting procedure, the authors selected a subset of 54 wearables for the purpose of this paper, yielding version 1 of the SiA-WD. In selecting this subset, we considered a wide variety of devices to obtain a database structure covering all aspects required for adding various devices (e.g., including different wearable types—e.g., watch, ring and patch—and covering the signals included in the database). We then focused on devices that combine various signals that have been repeatedly used in stress research and had relatively high usability or relatively high levels of established reliability/validity.

### Device information included in the SiA Wearables Database

For all devices, the database columns present relevant information on various aspects of the device and its measurement capabilities. A critical piece of information is the signals recorded by a device and the calculation of certain parameters. Accordingly, we report on both the actual signals (e.g., the columns of PPG, ECG, BP, Respiration) that are recorded and parameters that are provided using these signals. For the respiration column, it is important to note that a device is considered as measuring respiration signal (noted as 1 in the database) if it captures both the rate and the depth of breathing continuously. Example techniques would include thoracic and/or abdominal piezoresistive belts, strain gauge belts, respiratory inductance plethysmography, and impedance pneumography. Thus, a wearable only extracting respiration rate from the PPG signal would have a 0 in its respiration signal column, but respiration rate would be added as a parameter in the provided parameters column. Regarding the BP (blood pressure) column, all wearables that produce BP values are considered to measure BP and annotated accordingly in the database. The employed method is specified; e.g., cuff-based measures such as auscultatory and oscillometry are specified as well as cuffless methods based on predictive (machine learning) models.

To decide which other columns to add to the SiA-WD, we reviewed the extant literature on relevant criteria for the selection of a wearable. Existing recommendations (Byrom et al., 2018; Kleckner et al., 2021; Pantelopoulos & Bourbakis, 2008, 2010; Polhemus et al., 2020) demonstrate that a range of aspects should be considered before selecting a wearable. For example, the intended use of a wearable, be it clinical, research, or consumer, could be a selection criterion when assessing a patient population with a clinical grade device being required. Other general information, such as the price, is also essential, including one-time-purchase costs (e.g., the device and software) as well as additional monthly subscriptions (e.g., for application or data server use). Such aspects were grouped in the category of *general device information*. All technical information about the device was combined in the *technical specifications* category, including battery life or charging method and duration, which determines whether a participant can measure constantly during the study period or if they must charge or swap the device (Boateng et al., 2019; Liu & Han, 2022). Additionally, it is important to consider the availability of (raw) data and the form in which it is available, e.g., at the signal level or merely as time series of provided parameters. It is also useful to know where the data is stored, whether it is stored securely, and what software is required for data processing. These, among other data-related aspects, were grouped in the *data access* category (Kleckner et al., 2021; Siboni et al., 2016). The last set of columns contains information on *reliability, validity*, and *usability*, which are important criteria for selecting a wearable from a researcher perspective.

After having decided on these categories and their aspects, we fine-tuned the structure by actively seeking out the information for the first 10 devices in the SiA-WD. Based on the issues encountered, we added a number of aspects or changed how we reported on them. We extended this fine-tuning after consultation with experts on wearables research within the Stress in Action consortium. This led to the final column structure shown in Table 3.

**Table 3:** Device aspects included in the SiA-WD, grouped per category

Category	Column	Details
General device information	Device Manufacturer Website Release date Market status Main use Device costs Wearable type Location Size Weight	Name of the device Name of the manufacturer Link to the webpage of the device Official release date of the device on the market Upcoming/current/discontinued Research/consumer/clinical or their combinations One-time purchase price (EUR) and additional costs Type of the device (e.g., watch, CPU + electrodes) Location at which the device is worn (e.g., wrist, chest) Device dimensions in millimeters Device weight in grams
Signals	PPG ECG ICG EMG Respiration EDA BP Accelerometer Gyroscope GPS Skin temp Other signals	Photoplethysmography Electrocardiogram Impedance cardiography Electromyography Respiration Electrodermal activity (galvanic skin response) Blood pressure Accelerometer Gyroscope Global Positioning System Skin temperature All other signals the device can record
Technical specifications	Water resistance Battery life Charging method Charging duration Bio-cueing Bio-feedback	In terms of depth in meters and time in minutes Maximum battery life as specified by the manufacturer in hours Device charger or disposable/rechargeable batteries Time needed in minutes to fully recharge the battery Options to cue (e.g., vibration) users based on their physiology Access users get into their physiology (e.g., via device display)
Data access	Raw data available Provided parameters Sampling window of parameters Data transfer method Compatibility Required software Additional software Internal storage method Device storage capacity Server data storage GDPR compliance FDA approval/clearance CE approval/label	If signal-level data can be exported for analysis The parameters automatically generated by device Approximate time window around which each parameter is calculated, if available All methods of transfer (e.g., Bluetooth, SD-card) System compatibility of the device (mobile and PC) Software required to record and/or extract the data Additional (e.g., analytical) software available Internal storage availability and the method (e.g., SD-card) Hours and megabytes of data that can be recorded and stored internally Data stored on external servers (including their location) Compliance with the General Data Protection Regulation Act FDA [Food and Drug Administration] approval or clearance for the device or its components Device has been assessed to meet the European Union (EU) market regulations
Reliability, validity, usability	Highest level of validation evidence Number of validity and reliability studies reviewed Studied parameters General validity and reliability synthesis Number of usability studies reviewed General usability synthesis Hyperlink to the device RVU page	Highest level of validation available (external/internal/no validation) Number of relevant validity and reliability studies found in the search for a given device List of the parameters included in the reviewed RVU studies Short synthesis statement of the validity and reliability results of the reviewed studies Number of the relevant usability studies found in the search for a given device Short synthesis statement of the usability results of the reviewed studies Hyperlink to the separate RVU page with detailed reliability, validity, usability information for the device
Curators’ expert scores	SiA short-term usefulness score SiA long-term usefulness score	Average score based on the perceived usefulness of a device for the future short-term SiA studies as assessed by three raters Average score based on the perceived usefulness of a device for the future long-term SiA studies as assessed by three raters

**Fig. 2:**
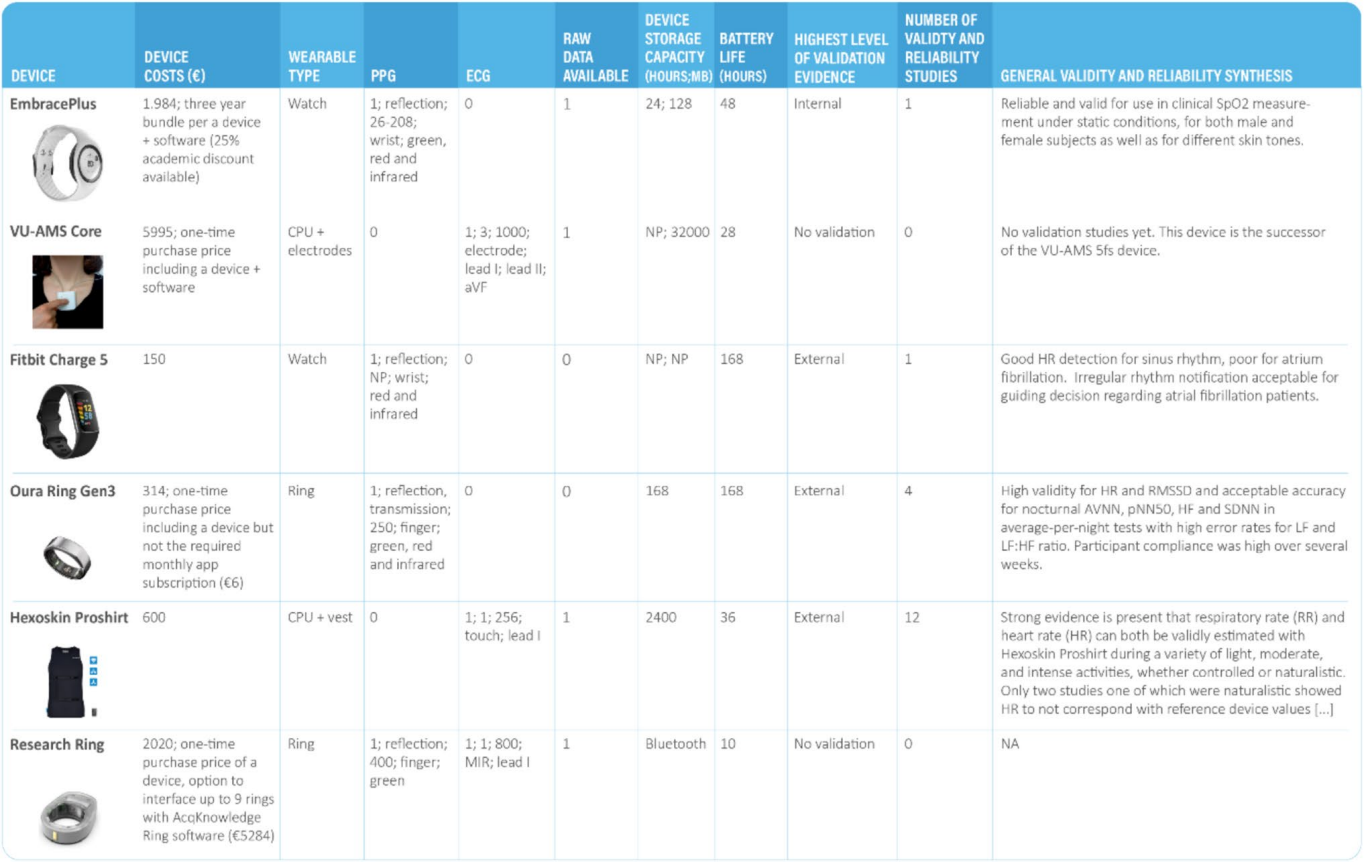
A section of the SiA-WD showing 11 of the 53 columns for six devices. *Note.* In these columns, 0 = not available, 1 = available, NP = not provided. The SiA-WD itself does not contain the images of the devices, but they are entered in this figure for illustrative purposes

The database contains a total of 53 columns. The columns represent the aspects relating to the five categories (general device information, signals, technical specifications, data access, reliability, validity, and usability). The last two columns include the SiA expert scores for short- and long-term assessment. As a brief illustration of the database, Fig. 2 shows 11 example columns for six different devices selected to represent the different categories of wearables (e.g., more research or consumer-oriented).

### Retrieving the relevant information for a wearable

To fill the cells of the database (i.e., retrieving the relevant information in each column for each of the selected wearables) for the general and technical device specifications as well as the information regarding the physiological measurement, we started by using the manufacturer’s website and device manuals in English. This meant searching the Web using the device name—and if known already, the manufacturer name—to find the manufacturer’s website and looking for the product specifics’ page and the device manual. In addition, we collected data from publications on studies that utilized the wearable. When any information on the relevant aspects was missing, a standardized email inquiring about the missing information was sent to the email address specified on the manufacturer’s website.

### Establishing the validity and reliability of devices in the SiA Wearables Database

Besides completing the *general device information, signal, technical specifications, and data access* columns, it is important to provide researchers with an understanding of the device’s measurement accuracy and consistency, that is, of whether a device measures what one expects it to measure and if it maintains doing so over extended periods of time. This can be sufficiently achieved by focusing on convergent validity and test–retest reliability (Hopkins, 2000). Convergent validity refers to how well a device’s measurements agree with that of a gold standard or other reference device (RD), under the same conditions while recording concurrently. Test–retest reliability assesses the extent to which the device, under similar conditions, produces the same results at different points in time (Kottner et al., 2011).

Assessment of convergent validity can be done at the signal, parameter, and event levels (van Lier et al., 2020). While we agree with van Lier and colleagues that wearables should ideally be validated at *all* these three levels, most validation papers to date do not report this extensive validation information. Furthermore, in nondiagnostic ambulatory monitoring, the raw time-series signal itself in general is not the focus of interpretation, but the parameters (e.g., HR, SDNN, RMSSD) derived from the signals frequently are (van Lier et al., 2020). Thus, when presenting validity information, our focus will be on convergent validity at the parameter level (e.g., to what extent RMSSD of a new wearable complies with that from a gold standard) and not, for example, on the signal-to-signal cross-correlations. For the current version of the database, we did not include studies that investigate a wearable’s construct validity (e.g., whether parameters such as heart rate indeed differ between baseline and intense exercise) or ability to make predictions using machine learning models (e.g., predictive validity of the PPG signals for subjective stress detection).

### Establishing usability of devices in the SiA Wearables Database

Apart from validity and reliability, the research value of a wearable also depends on its usability, as this is a main contributor to participant compliance in ambulatory studies. High usability can also enhance optimal use by participants and thereby increase signal quality and reduce data loss. Among participants who initially agree to put on wearable monitors for a longitudinal study, the majority may remove it before study termination due to getting irritated, uncomfortable, overwhelmed, or unwell (Jeffs et al., 2016), and may perceive the monitor as impeding their usual activity (Areia et al., 2020; Ehmen et al., 2012). Dias and colleagues (2009) furthermore asserted that skin reactions may occur with certain wearables clenched tightly onto the skin. These can result not only in a reduction of quality data but also in ethical and medical concerns. Thus, to get an understanding of usability (including user-friendliness and user acceptance), we identified papers that conducted quantitative or qualitative (e.g., posing systematic questionnaires or open interviewing) research on these aspects.

### Compiling the reliability, validity, and usability information of devices

The reliability, validity, and usability of the wearables were established by a literature search of papers on *original* experimental studies using the wearable (i.e., not meta-analyses or reviews) that were published in English in peer-reviewed journals or conference proceedings. No filtering was performed based on the year of publication. As the search strategy was to include all papers that investigated a given device’s reliability, validity, or usability, the search string included terms relevant to all three aspects along with the device name: “((Device Name) AND (valid* OR reliab* OR compar* OR accur* OR verif* OR usab* OR"user experience"OR"user friend*"OR user-friend*).” If a device had multiple names for the given version, they were all added as a device name using the OR operator.

Papers were only considered to supply the necessary validity information when they mentioned the use of a reference device (RD) in their abstract, such as the following: “Measurements were recorded simultaneously using the Hexoskin and Polar Team Pro” (Haddad et al., 2020). Furthermore, the RD had to be a proven gold standard (i.e., showing greater established precision than the wearable being validated), such as the COSMED K5 for respiratory volumes or an ambulatory device consistently known to be accurate for heart rate, such as the Polar H10 Band. If two wearables were merely being compared to one another without a clear rationale for one of them to be considered as the golden standard/ground truth, the paper was excluded. See Table 4 for a list of eligibility criteria for papers to be accepted as providing device-specific information.

**Table 4:** Eligibility criteria for device-specific studies on validity and reliability

Inclusion criteria	Exclusion criteria
• Assessment of parameter-level convergent validity, test–retest reliability, and/or usability • Assessment of convergent validity • Peer-reviewed articles and conference proceedings published in English	• Studies on construct validity only • Studies on machine learning-based detection of secondary outcomes (e.g., perceived stress) • Meta-analyses and reviews • Theses, gray literature, other text that was not peer reviewed

### Increasing the efficiency of the literature search

**Fig. 3:**
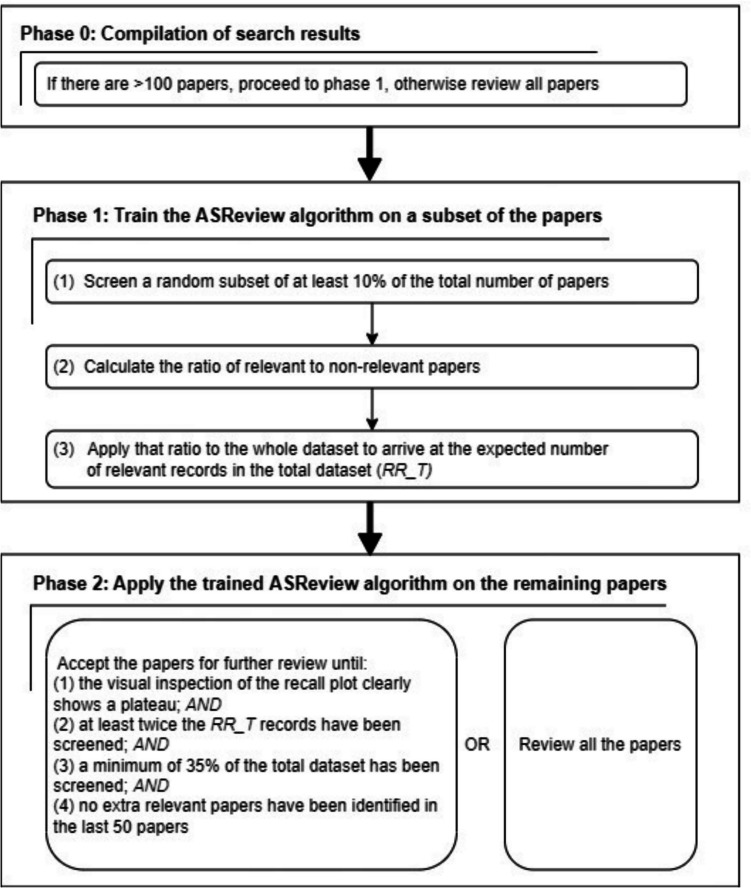
Using ASReview to select papers for a detailed review of reliability, validity, and usability of a wearable

For several wearables, the literature review produced a large number of hits (e.g., 530 papers for Empatica E4 device). Given the rapid expansion of this literature, this challenge protracts into the future maintenance of the database. We therefore developed a procedure to efficiently extract relevant papers, using the ASReview version 1.6.2 (https://asreview.nl/) screening tool, used when there were more than 100 papers found for the search for a device. ASReview can greatly reduce the time needed to select the relevant records through a so-called active learning method, in which a machine learning model continuously rearranges the items based on the decisions made by a researcher regarding their relevance (Van De Schoot et al., 2021). The two phases of the literature review were based on the SAFE procedure (Boetje & Van De Schoot, 2024) and are shown in Fig. 3. Further details on the approach can be found in Supplementary 1.

### Data extraction of the RVU (reliability, validity, usability) information

For each wearable in the database, detailed information on reliability, validity, and usability were extracted manually from the papers identified. This information was collated in device-specific worksheets referred to as the RVU worksheet and were maintained separately from the database. RVU represents the reliability, validity, and usability information of a device, as extracted from the papers identified in the search. A direct hyperlink to these device-specific RVU worksheets is provided in the database. Reliability and validity data were extracted at the level of the provided parameters (e.g., heart rate), as papers can focus on different parameters from the same device and can report differential validity and reliability performance for these parameters. The information extracted for the RVU per device per parameter is listed in Table 5. Below, we first explain which data is extracted per paper and then clarify how the information of all papers for a device (as found in the RVU worksheet) is used by the SiA-WD curators to create the synthesis statements in the database.

**Table 5:** Data extraction form for reliability, validity, and usability of each device, gathered in the device-specific information page

Data category	Column name	Description
Metadata	Study citation	In APA 7 th edition format
	Year of publication	YYYY
	Publication journal	Journal name
Study characteristics	External/internal	Was the study performed internally (i.e., by device manufacturers) or externally
	Population	Population type (e.g., elderly)
	Sample size	Number of participants
	Age	Mean and SD
Methods (reliability, validity, and usability of the device)	Reference device	The gold-standard reference device used for comparison, e.g., standard clinical ECG
	Number of events	Number of tasks included
	Included events	Types of tasks included
	Studied parameters	All the parameters studied (e.g., HRV and SCL)
	Time between test–retest	Time after which authors made a second measurement with the device under same circumstances to calculate internal consistency
Findings and conclusions	Authors’ conclusion	Excerpt of authors’ (who empirically assessed the device) conclusion
	Validity and reliability summary	Summary of validity/reliability findings written by the curators
	Usability summary	Summary of usability findings written by the curators
	Final verdict (negative/neutral/positive)	Curator’s final verdict per physiological parameter based on all available RVU information

From each paper of a device, first, the overall conclusion of the authors who evaluated the device reliability, validity, and/or usability were extracted as direct quotations (e.g., “Hexoskin was able to correctly measure tidal volume in healthy subjects during various tasks […]"by Mannée and colleagues (2021)), typically taken from the abstract. This column in the RVU worksheet was called the “Authors’ conclusion,” referring to the authors who empirically tested the device, and not to the curators (the three first coauthors of the current paper). If the test–retest reliability was also examined in a study, the authors’ conclusion also included the reliability statement per parameter tested. If specified, the time interval between two testing points was entered into the RVU worksheet as a separate column.

Apart from the authors’ conclusion on convergent validity and test–retest reliability, information on the independence of the validation work, the reference device used, and the scope of conditions and populations considered in the validation studies were entered (see Table 5*Metadata*, *Study characteristics* and *methods*). We determined the nature of a study, external or internal, by reviewing conflict-of-interest disclosures. External validations are those conducted independently from the device manufacturers and typically show less bias than internal studies. In line with the INTERLIVE consortium (Johnston et al., 2021; Molina-Garcia et al., 2022; Mühlen et al., 2021) that proposed recommendations for systematic validation of commercial devices, we argue that variables including the sample size, type of population, duration of data collection, and the testing conditions (i.e., tasks and the presence of daily life components) matter in determining the trustworthiness of a validity study. The relevant information was located by scanning the full text of papers and entered in the RVU worksheets to help future researchers using the database assess if a device has been sufficiently validated for the tasks, populations, and measurement durations of their interest.

The authors’ conclusion may sometimes lack important methodological information on the study. Thus, after reviewing a given paper, curators wrote a “Validity and reliability summary” and a “Usability summary” whenever applicable. The summaries aim to succinctly capture the most important methods, findings, and unique sample characteristics in a paper. An example “‘Validity and reliability summary” is “[...] The 95% Limits of Agreement were − 3.89 to 3.77 (mean bias 0.06) beats per minute for HR and − 173 to 171 (mean bias − 1) for IBIs. Results were comparable across all subgroups (i.e., different skin type, hair density, age, BMI [body mass index] and gender).” An example usability summary is"87% of participants successfully and consistently wore the ring. Gender, age and weight did not influence the adherence. 56% were willing to continue wearing the ring after the study […].” After considering all information extracted from a paper, curators made a final verdict for each studied parameter: positive, neutral, or negative (e.g., HR: positive; RMSSD: positive; HF-HRV: negative).

### Synthesis of reliability, validity, and usability studies for the SiA Wearables Database

After completing the review of the RVU studies for a device, a synthesis statement for validity and reliability was written in the SiA Wearables Database by the curators. This synthesis combines all the available validity and reliability studies in the device-specific RVU page—and another for usability—combining all the usability studies in the device-specific RVU page. An example excerpt of a general validity–reliability synthesis is as follows: “Most studies comparing E4 to a reference device (RD) suggest high validity of HR under static and dynamic conditions (one study found opposite results) and often report HRV parameters as valid but only in static conditions, although results seem conclusive only for RMSSD and SDNN […].” An example usability synthesis is “Hexoskin is perceived by participants to be highly comfortable when used in a laboratory procedure. It can be used in those with chronic obstructive pulmonary disease […] Use in an ambulatory study of a week, however, is problematic […].” Such synthesis statements allow researchers to review all validation or usability conducted for a device. Thus, they can make expedited and systematic between-device comparisons in reliability, validity, and usability. In addition to the validity–reliability and usability syntheses, key information from the RVU worksheet of a device was also entered into the SiA-WD. The full account of these columns is listed in Table 6.

**Table 6:** Reliability, validity, and usability information on the wearable in SiA-WD

Column name	Entry type	Explanation
Highest level of validation	No validation/internal/external	External validation ranks higher than an internal one
Number of validity–reliability studies reviewed	Numeric	Number of validity and/or reliability studies resulting from the search
Studied parameters	List	All the parameters present in all the studies
General validity and reliability synthesis	Text	A comprehensive but short written overview of all the different study results. This includes both validity and reliability information
Number of usability studies	Numeric	Number of usability studies resulting from the search
General usability synthesis	Text	A condensed overview of the device usability based on the reviewed studies. This includes not only user experience but also aspects such as adherence

### Wearable selection for applications in stress research

After populating the SiA-WD, the curators created a scoring system to rank order the wearables based on their usefulness for stress research. In this scoring, the curators were strongly guided by the perceived usefulness of the wearables for the future large-scale cohort studies within the Stress in Action project. These cohort studies seek to answer questions on both short-term and long-term relationships between physiological, emotional, cognitive, and behavioral responses to stress in within-person studies and the predictive value of these responses for disease outcomes in between-person studies. Within-person studies require long-term assessment across weeks or months of at least one physiological indicator of stress in the same participant, whereas between-person studies to predict disease require more intensive measurements of physiological stress systems—i.e., measuring various signals and output parameters—for one to three full 24-h recordings, possibly on selected days of the week. While a high level of validity and reliability is always essential independent of wear time, high levels of usability become increasingly more important for longer wear times. Accordingly, two separate scores were given by the three curators to each device reflecting its perceived research usefulness for both short (i.e., approximately 2 days) and long (i.e., at least 2 weeks) measurement durations.

Since scoring the devices is an inherently subjective process, a set of criteria had to be outlined to achieve standardization of the rating by the three curators. The criteria list came from the columns in the database (e.g., raw data availability, validity, usability). Separate scoring criteria were used for a typical short-term (approximately 48 h) and long-term (2 + weeks) ambulatory study. The finalized scoring criteria per term of use is listed in Table 7. Blinded to each other’s scores, each curator scored each device on a scale of 1 (least optimal) to 10 (most optimal) for both short-term and long-term use. The scores of the three curators were averaged into the final expert scores for short- and long-term studies. These were included in the database. No score was entered for devices that had been on the market for less than 8 months. This avoids unfair scoring of devices which were just released on the market, for which little to no external validation research will be available.

**Table 7:** List of scoring criteria ordered by their importance for short-term and long-term studies

Short-term importance of criteria		Long-term importance of criteria	
• GDPR approval	High	• GDPR approval	High
• CE approval	High	• CE approval	High
• All reliability– validity criteria	High	• Price	High
• Number and type of different signals that can be measured by the device (e.g., PPG, ECG, accelerometer)	High	• Number of different physiological parameters that can be measured by the device (e.g., HR, Skin temperature)	High
• Raw data availability	High	• Wearable type, location, and weight and size	High
• Provided parameters	Medium	• Usability outcome (if available)	High
• Parameter sampling window	Medium	• Battery life	High
• Price	Medium	• All validity-reliability criteria	High/medium
• Wearable type, location, and weight and size	Medium	• Parameter sampling window	High/medium
• Usability outcome (if available)	Medium	• Data storage capacity	High/medium
• Data storage capacity	Medium/low	• Data transfer method	Medium
• Data transfer method	Medium/low	• Data Storage Method	Medium
• Data storage method	Medium/low	• Charging duration	Medium
• Battery life	Low	• Charging method	Medium
• Charging duration	Low	• Raw data availability	Medium
• Charging method	Low	• Data Transfer Compatibility	Medium
• FDA approval	Low	• Waterproof	Medium/low
• Waterproof	Low	• Bio-cueing	Medium/low
• Bio-cueing	Low	• Biofeedback	Medium/ow
• Biofeedback	Low	• FDA approval	Low

Many devices regularly produce updated versions. This is especially true for consumer devices which are often upgraded for commercial purposes with the focus on aesthetics or usability changes. For newer versions that did not introduce new signals or a different form factor, we used the information on the previous version of the same line of product to guide the scoring. For example, studies on the Fitbit Charge 4 could guide the scoring for Fitbit Charge 5, but the Fitbit Sense cannot be used in the scoring of the Fitbit Charge 5. It was assumed that in the absence of large changes to its hardware or software (although this was a subjective judgement of the curators), the validity and reliability findings on the previous version of a given device would be indicative of the validity and reliability of the newer version.

## Results

### Descriptives of wearables included in the database

#### General device information

The first version of the database contains 54 devices that were primarily intended for consumers (35), followed by research (18) and clinical use (10), including nine devices intended for multiple user fields (e.g., research and clinical). The average price of a wearable is €1,013 with substantial differences in device costs (*SD* = €1,378) caused by a big gap between prices of consumer and research/clinical devices. Wearables intended for consumers cost on average €347 (*SD* = €175), whereas prices of clinical- and research-oriented wearables average out to €1,489 (*SD* = €1,403) and €2,082 (*SD* = €1,713), respectively. For six wearables, all intended for clinical settings, the price is only available on request. Moreover, 11 out of 54 devices have additional software or subscription costs. The most common type of wearables included in the database is a *watch* (40.7%) followed by a *ring* (16.7%) and *CPU with external electrodes* (16.7%). Other form factors were straps—sometimes combined with a CPU—worn on the upper arm, wrist, or with flexible positioning possibilities. The location where most wearables are worn is the wrist (44.4%), followed by finger (18.5%) and chest (18.5%).

#### Signals

Regarding the physiological signals, those most often measured by the devices were PPG (64.8%), ECG (50.0%), skin temperature (35.2%), blood oxygen saturation level (SpO_2_) (31.5%), and EDA (31.5%). The respiration signal, derived from either ICG or respiration belts, is measured by 5.6% of the devices. For some physiological parameters it is necessary to record multiple physiological signals. For example, to obtain peak–valley RSA, both an ECG and a respiration signal are needed. Among devices, 79.6% are equipped with sensors for multiple physiological signals. All three devices which record ICG measure ECG simultaneously.

Of the co-recorded signals that can account for confounding by posture, physical activity, and ambient noise level and temperature, accelerometry was the most common (90.7% of the devices). A further 20.4% of devices also offered GPS functionality, through a built-in sensor in the device itself. A total of 37.0% of the wearables included a gyroscope. Among other signals identified, the most common one was SpO_2_, with 31.5% of devices providing this option, followed by ambient light detection in 25.9% of the devices.

#### Technical specifications

A total of 83.3% of devices were water-resistant. The average battery life was 261 h for continuously measuring devices, ranging from 10 h for the BIOPAC Research Ring and Plux respiBAN BLE to as long as 30 days for the Withings ScanWatch 2. The majority (83.3%) of these wearables have a battery life of at least 24 h.

#### Data access

All devices except two have internal storage capacity, with options to transfer the data either through Bluetooth (92.6% of the wearables), via a (micro-)SD card (7.5%) and/or using a cable (16.7%). Only the BIOPAC Research Ring and Cosinuss c-med° alpha do not have any internal storage capacity and utilize direct Bluetooth transfer of the data to the manufacturer's application, therefore requiring continuous connection between the wearable and the phone.

Regarding the data output, 35.2% of the devices provide raw data, all intended for clinical or research purposes. Among the consumer-oriented parameters provided by the devices, the most common ones are heart rate (92.6%), sleep staging (75.0%), and physical activity scores (64.8%). “Stress” as a separate parameter is provided by 33.3% of the devices, commonly referred to as *stress management score*. A parameter that represents essentially the same might be named differently by manufacturers. To illustrate this issue, physical activity-related scores fall under the following names: *activity counts, hourly activity, activity score, active time, active zone minutes, exercise tracking, activity detection,* and simply *activity.* Even after contacting manufacturers for more detailed information on the provided parameters, we did not obtain the information needed to standardize the terminology. We, therefore, opted to include the names of parameters provided by the manufacturer.

#### Missing data

Twenty-four columns contain all the required information about all devices. However, not all information is provided by the manufacturers at all instances, even after requesting this information through email. This resulted in 284 NP (i.e., not provided by the manufacturer) values in the database. On average, columns contain 5.9 NP values, and most frequently, the sampling rates of consumer wearables were not provided. After missing sampling rates, the column of the *Device storage capacity* contains the highest number of NPs (49). For many devices (22), details on their water resistance level are missing, and if provided, lacking standardization between manufacturers in the way this information is reported.

### Reliability, validity, and usability

Of all devices combined, there were 96 papers in the database regarding reliability and validity, and 18 for usability. The Polar H10 had the highest number of relevant reliability and validity papers (17), and the Empatica E4 had the most usability papers (5). For 31 devices, no reliability, validity, and usability papers were identified. Twenty-two of the 54 devices had external validation, and three devices had only internal validation.

### Curators’ expert scores

Inter-rater reliability of the perceived usefulness of the wearables for the future large-scale cohort studies within the SiA project was calculated between all curators. The inter-rater agreement between each pair of curators was calculated via Pearson’s correlation. Using the *psych* package in RStudio, the two-way random effects intraclass coefficient ICC (2, *k*) was also calculated to represent the absolute agreement between all three curators’ scores (Koo & Li, 2016). Three out of the 54 devices in the database were not scored, as 8 months have not passed since their release date. The short-term and long-term usefulness scores were provided for 51 devices. The average ICC for long-term usefulness across three curators was 0.85, 95% CI = [0.76, 0.91], *F*(50, 100) = 6.6, *p* < 0.001. For the long-term usefulness scores, Pearson’s *r* was 0.58 between rater 1 (Saygin) and 2 (Schoenmakers), 0.72 between rater 1 and 3 (Sikora), and 0.68 between rater 2 and 3. The average ICC for short-term usefulness across three curators was 0.87, 95% CI = [0.78, 0.92], *F*(50, 100) = 8.0, *p* < 0.001. For short-term usefulness scores, Pearson’s *r* between curator 1 and 2 was 0.59, between curator 1 and 3 was 0.76, and between 2 and 3 was 0.77. Statistically significant and good (Koo & Li, 2016) absolute agreement was present across curators in both the short- and long-term usefulness scores of wearables.

The device with the highest short-term usefulness score was the VU-AMS 5 fs (8.7 out 10), followed by movisens EcgMove 4 (8.0), a tie between Hexoskin ProShirt and VU-AMS Core (7.7), and MindWare Mobile, Plux BioSignal kit, and Polar H10 (all three scoring 7.0). Devices with a moderately high score included the ambulatory blood pressure monitors Novacor Diasys 3 plus (6.3) and Spacelabs OnTrak (6.7), as well as BioHarness 3.0 (6.5) and Equivital Eq. 02 + Lifemonitor (6.8). All other devices scored less than 6 on short-term usefulness. The highest long-term use score of 8.0 was achieved by Empatica EmbracePlus and Fitbit Sense 2. They were followed by Polar H10 and Garmin vivosmart 4 (7.5), Fitbit Sense with 7.3, Oura Ring gen3 with 7.3, and Garmin vivosmart 5 with 7.0. There were a number of devices that had moderately high scores for long-term use, including Garmin vivoactive 5, WHOOP 3.0, Withings Scanwatch (all with 6.8), Empatica E4 (6.7), Fitbit Charge 5 (6.7), Corsano Cardiowatch 287–2 (6.7), WHOOP 4.0 (6.7), Google Pixel Watch 2 (6.3), Garmin vivoactive 4 (6.3), Corsano Cardiowatch 287–1 (6.0), Apple Watch Series 8 (6.0), and NOWATCH (6.0).

### Illustration of the use of the SiA-WD in two different stress research scenarios

Depending on the aim of a given ambulatory study, human physiology can be recorded for durations ranging from several hours to years. In addition, the type and combination of parameters, along with the underlying signals included in different ambulatory studies, vary widely. Numerous other criteria including raw data extraction, availability of provided parameters, and costs can also be fundamental decision points. Although many wearables are available for research, they may be unsuitable after considering the study plan, duration, resources, hypotheses, and theoretical framework. Our Stress in Action Wearables Database provides detailed information to assist in these decisions. Below, we present two different research scenarios, one for a short-term (Research Scenario A) and one for a long-term study scenario (Research Scenario B) on the activity of physiological stress systems in daily life. We first illustrate the steps one might take in using the SiA-WD to get to a shortlist of wearables that fulfill the study requirements and then review which information in the overall database as well as the RVU worksheets might be used to make a final decision. Nonetheless, researchers might also benefit from using the database in a more pragmatic manner, for instance, to check how a device they already have, or a recommended one, performs in comparison to other wearables. Therefore, device-based comparison is facilitated by the online filtering tool, but a more structured approach to device selection, as illustrated by the scenarios below, is recommended to best support the study purpose and its design characteristics.

#### Research Scenario A: Short-term study of how cardiovascular threat-challenge response patterns impact health outcomes

Research Team A is interested in the impact of perceived threat and challenge in response to daily life stressors on cardiovascular health outcomes. Informed by the threat-challenge hypothesis (Blascovich & Tomaka, 1996; Tomaka et al., 1993), the team seeks to continuously record left-ventricular contractility (indexed by the pre-ejection period, PEP), cardiac output (CO), and total peripheral resistance (Wormwood et al., 2019). According to this hypothesis, a threat should be characterized by a dominant vascular response (increased total peripheral resistance), whereas challenge generates a cardiac response (shortened pre-ejection period, increased cardiac output). Thus, ambulatory impedance cardiography plus electrocardiography are required in conjunction with a cuff-based ambulatory blood pressure monitor. The main goal for this research scenario is disease prediction, and so it has a largely between-subject design. Specifically, the researchers want to investigate whether the effect of the differences of trait anxiety (as assessed by a validated questionnaire) on the health outcome of cardiometabolic/immunologic “allostatic load” risk profile (as assessed by future blood sampling) (Robertson et al., 2017; Seeman et al., 2001) is mediated by a predominance of cardiovascular threat reactivity in daily life. They aim to measure perceived threat and challenge via mobile ecological momentary assessment. As a secondary research question, Team A is interested in whether physiological levels measured during sleep, in particular of cardiac vagal control, are linked to threat or challenge reactivity in daily life (Mendes et al., 2001). To index cardiac vagal control, they seek to use respiratory sinus arrhythmia (de Geus & Gevonden, 2024).

A total of 110 participants with moderate generalized anxiety disorder are scheduled to be measured within 30 weeks. Each participant’s recording will take place over 2 days within the same week: one working and one leisure day, including sleep. As the participants will return to the lab in between their recording days for device replacement, a minimum battery life of 24 h is needed. High reliability and validity of the physiological measurements under naturalistic conditions are considered crucial. They have a budget of **€**14,000 for purchasing devices. The researchers have a strong preference for retrieving raw data to derive the parameters themselves. See Table 8 for an overview of the requirements for Research Scenario A.

**Table 8:** Description of the requirements for Research Scenario A

Requirements for Research Scenario A	
• Signals	Raw ICG and ECG data (optional respiration signal)
• Parameters	PEP, RSA (optional RR), HR, BP, CO, TPR
• Participants	110 anxiety patients
• Project duration	30 weeks
• Assessment duration per participant	2 days within a week
• Maximal costs per device	**€**6,000
• Minimum battery life	24 h
• Validity & reliability	High

After opening the SiA Wearables Database tool to find suitable devices, they needed to decide between selecting either the *signals* (e.g., PPG or ECG, ICG, and BP) or the *parameters provided by device* (e.g., CO, PEP, BP, RSA, RR) for the first stage of filtering. The researchers opted for providing a list of signals they require a device to have, as they wanted to extract the needed parameters—CO, total peripheral resistance, PEP, RSA—using their own data-analytic software rather than obtaining them directly from the device. In this case, the device would need to have ECG and ICG and, if possible with the same device, also the ability for BP recording. Because thorax impedance signals also provide a respiration proxy signal (dZ), it can be used in combination with the ECG to calculate peak–valley RSA. Therefore, the researchers do not specify respiration as an additional required signal, but select devices that simultaneously record ICG, ECG, and BP.

The SiA-WD contained no devices that can simultaneously measure the ICG, ECG, and BP. The researchers, therefore, searched the database anew, now looking for a combination of two devices, one measuring ECG/ICG and one measuring cuff-based ambulatory BP. Their budget allows them to buy a minimum of two of the same devices that record the ICG and ECG and two that record blood pressure. This aligns with the logistic capacity and time frame of the study (four people per week to be equipped for 2 days). Consequently, ICG and ECG as required signals, 24 as the minimum battery life, and €6,000 as the maximum device cost are entered (€2,000 are set apart for BP monitor costs to be used in the second search). As it will be a short-term study, they decide against filtering based on the form of the wearable (e.g., not restricting to wrist placement). Choosing to view their results sorted by the devices’ SiA-expert score on short-term usefulness column (alternative sorting options are available such as based on the number of validity studies or device cost), they obtain the following three devices, listed from the one highest to lowest scoring: VU-AMS 5 fs, MindWare Mobile, VU-AMS Core.

Next, the researchers examine the raw data availability of these three devices in the database. They all allow raw data extraction. Then, each device’s overall reliability–validity conclusions are read, and the device-specific RVU sheet (consisting of all reliability, validity, usability papers of a device and detailed information) is investigated to understand if the parameters of interest were validated for a given device, along with the specifics of such validation. As they see VU-AMS 5 fs was already shown to be valid for all parameters of interest, is CE-approved, and has a comparatively lower cost including the data analysis software, it becomes the device of choice. They then perform a search for only ambulatory blood pressure monitors that are in-market, entering “BP” to the signal required without further filtering and obtain Novacor Diasys 3 plus, Garmin Index BPM, and SpaceLabs On Trak. As the Garmin blood pressure monitor is the only one matching the budget criteria, is FDA-cleared, and has some validation, they decided on using the Garmin Index BPM in conjunction with the VU-AMS 5 fs.

#### Research Scenario B: A long-term study on the relations between physical activity and physiological stress-reactivity

Research Team B is interested in testing the cross-stressor adaptation hypothesis of physical activity in the daily life context. The cross-stressor adaptation hypothesis posits that regular exercise leads to adaptations in stress response systems that leads to reduced physiological reactivity in response to psychological stressors (von Haaren et al., 2016). Current empirical evidence has been mixed, and no studies have conducted a prolonged assessment of the relations between physical activity and physiological stress-reactivity in daily life (Van Der Mee et al., 2023). The researchers are mainly interested in the effects of moderate-to-vigorous activity periods, which typically occur with low frequency in a general population, on stress reactivity. Regular exercisers are expected to harvest a larger benefit in terms of more strongly attenuated stress-reactivity after a period of moderate-to-vigorous activity than non-exercisers. In short, they seek to record physical activity and physiological reactivity across a longer period of 3 months in a sample of at least 300 participants, with the focus on the within-subject relations between physical activity and physiological stress-reactivity.

The default method to assess stress levels in daily life is ecological momentary assessment involving repeated self-reports by smartphone beeping, which is burdensome. Alternatively, this stress response can be measured via physiological reactivity after accounting for periods of activity. Accordingly, the researchers use the “additional heart rate” approach (Brouwer et al., 2018). In this approach, increases in heart rate are flagged only when co-recorded physical activity indicate that the heart rate response was not simply part of homeostatic regulation in response to changed hemodynamic and metabolic demands (Verkuil et al., 2016).

Therefore, they require a wearable that can make prolonged recordings of physical activity (measured by an accelerometer) and measures of physiological arousal, like heart rate and electrodermal activity (optionally HRV). Although the reliability and validity of the physiological measurements are important, it is crucial to have many recording days within a single person. As participants will be tracked over 3 months, they only allow wearables in the form of rings and smartwatches to increase adherence. The wearable should give a minute-by-minute index for physical activity (e.g., vector magnitude or the number of steps) and for general physiological arousal (5-min epoch with an increase in HR or ns.SCR of more than 20% compared to the previous epoch). See Table 9 for an overview of the requirements for Research Scenario B. The researchers filter based on the parameters provided by the device. Considering that combining increased HR and increased ns.SCR may improve estimation of the physiological arousal level, they opt for selecting devices that can readily provide *both* parameters for at most 5-min time windows along with physical activity-related parameters. As the researchers think having the participants charge their wearable every 2 days is a reasonable frequency, minimum battery life is set at 48 h, and the resulting devices were sorted based on their SiA long-term usefulness scores, from highest to lowest scoring, resulting in the following: Fitbit Sense 2 (8 points), Empatica EmbracePlus (8), Fitbit Charge 5 (6.7), and NOWATCH (6). All devices provide distance, steps, and active zone minutes parameters, which can be used to index physical activity. The details of the SiA-WD show the Fitbit Charge 5 does not provide continuous but manually initiated scan-based values, which does not suit the requirements and is therefore excluded. Similarly, NOWATCH measures EDA but does not provide parameters derived from the signal. Empatica EmbracePlus has one validity study, which only examined the blood oxygen saturation levels. Fitbit Sense 2 lacks reliability, validity, or usability studies. When checking the general validity and reliability synthesis column, it is seen that although Sense 2 does not have validation studies, its predecessor device Fitbit Sense’s HR was found to have overall acceptable validity under both static and active conditions, and its skin conductance level had a significant positive correlation with the reference device parameters. Because Fitbit Sense 2 is defined as a successor of this previous release, the researchers assume the latest version to perform up to similar validity standards as the previous version. As the study will be carried out in Europe, they check the GDPR column to make sure the device’s way of storing data meets the data protection regulations required by law and choose Fitbit Sense 2 for the study.

**Table 9:** Description of the requirements for Research Scenario B

Requirements for Research Scenario B	
• Signals	PPG or ECG, and accelerometer
• Parameters	HR, ns.SCR, and physical activity-related parameters (optional HRV)—provided at least every 5 min
• Participants	≥ 300
• Assessment duration per participant	3 months
• Minimum battery life	48 h
• Usability	High

## Discussion

Selecting the right wearable for stress research can be challenging in face of the large number of physiological wearables on the market paired with the lack of a comprehensive, systematic, and iteratively updated overview of the relevant wearable characteristics (Connelly et al., 2021; Dobson et al., 2023). The Stress in Action Wearables Database (SiA-WD) provides such an overview, enabling stress researchers to compare a host of wearable devices on a number of research-informed characteristics with detail. Compared to other overviews of wearable devices such as systematic reviews and databases (Iqbal et al., 2016; Lu et al., 2023; Paredes et al., 2021; Vijayan et al., 2021), the SiA-WD has several major advantages. First, the database is comprehensive regarding the information on general device information, technical specifications, and data access. Second, the technical, practical, and importantly, physiological details were obtained with a *systematic* review including the reliability and validity of the measured physiological parameters. This is—to the best of our knowledge—a first in the field. Third, where other overviews may have a specific focus, such as the focus on cardiovascular parameters by Lu and colleagues (2023), the SiA-WD has a broad scope of signals and devices relevant for physiological research focusing on autonomic nervous activity. Thus, the database applies to a relatively larger audience of researchers in the field. Fourth, this systematic search included a usability assessment also with a systematic search of studies that might have investigated its user-friendliness. The curators’ summary statements on reliability, validity, and usability papers make an easy and time-efficient selection of devices possible, while the device-specific reliability, validity, and usability (RVU worksheets) allow access to the evidence supporting these summary statements. And fifth, where other overviews get outdated, the SiA Wearables Database will be consistently extended and updated.

### Challenges encountered

Populating the database was not without its challenges. As some manufacturers did not provide the required information, they were sent a standardized email requesting these details. Most often, information on the particulars of physiological sensors, a comprehensive list of parameters provided by the device, and the sampling windows of parameters (e.g., the number of seconds over which each heart rate is calculated) were lacking. In the case that no response addressing the question(s) was received, information fields had to be filled with NP (not provided by the manufacturer). For example, columns specifying the signals’ sampling frequency or the device storage capacity contained frequent NPs, because this information could not be retrieved. Another challenge in populating the database concerns the lack of clear definitions of the output parameters of the wearable systems. For example, heart rate can be calculated continuously per 5-min intervals, per day, per activity, and so on, but the used time scale was not always available for data extraction. Moreover, upon filling in the database, it became apparent that researchers conducting long-term ambulatory studies may use external applications as a solution, as it facilitates more detailed data extraction and storing of the recorded signals and parameters; e.g., the Heart Rate Variability Logger application. Finally, conceptually similar parameters go by many different names, as exemplified by the heterogeneous terminology used for physical activity.

The SiA Wearables Database version 1.0 includes more devices that are consumer-grade rather than research-grade devices. This probably explains why more than half of the wearable systems do not provide raw data access and instead focus on providing parameters such as HR, sleep stages, stress scores, and activity values. Wristwatch was the most used wearable type, and ECG and PPG were the signals most often recorded, with most devices also co-recording accelerometry. A watch is a convenient and generally accepted way of wearing a device, and the integration of PPG sensors and accelerometers can provide parameters (e.g., HR values and activity scores) that are informative for a broad audience. Due to its relatively lower burden (for example, as compared to a vest), it also enables higher adherence and comfort for populations with higher risk factors. Consumer-grade wearables are substantially cheaper and more user-friendly than research-grade wearables. Compared to research-grade wearables, consumer-based wearables are therefore much more suitable for research questions that need to assess many people and/or for an extended period.

However, many parameters produced by consumer devices lack validation, as shown by the overview of the validity and reliability syntheses. In terms of established validity and reliability, research-grade wearables clearly outperform consumer wearables. Research-grade devices also more often provide raw data and record multiple different signals, such as continuous ECG and ICG. A full continuous ECG is rarely recorded in consumer wearables, and those that record ECG do so by having the user manually initiate a short ECG recording by placement of the hand on top of the device (e.g., to the side of a watch). Furthermore, the participants are typically asked to refrain from any physical movement which might create stress because of multiple failed recordings (Seshadri et al., 2020). A research-grade device in a low-burden wearable format such a wristwatch could have high utility for research interested in assessing autonomic and physical activity parameters for more fine-tuned windows and over longer periods of time.

The results highlighted the lack of both validation and usability research on existing wearables, as well as the need for a more systematic approach to such studies, especially when one considers the high tempo with which devices are discontinued and introduced to the market. Although systematic validation studies are performed for some wearables, the reported statistics vary widely (e.g., Bland–Altman plots, intra-class correlations, linear regression, half-split reliability), which complicates comparisons between studies and across devices. This constitutes a challenge for both the curators and users of the database in making a fine-tuned comparison between those devices that are deemed valid. We hope that the gaps in validation studies identified by the SiA Wearables Database will create incentives for studies assessing the reliability, validity, and usability of wearable technology in a more consistent manner with regard to study design and statistical reporting (Keogh et al., 2021; Liang et al., 2018; Shei et al., 2022; van Lier et al., 2020).

The two selected research scenarios where the SiA Wearables Database is used illustrate the need for such a database. There is rarely a clear “winner” device that fits a study design. Instead, the optimal device choice depends on a consideration of many aspects of the research study, including the theoretical relevance of the physiological parameters to be measured, the total number of participants, the duration of the sampling, the burden threshold that can be tolerated by the participants, and financial and logistical constraints of the research team. Filtering by the relevant columns in the SiA-WD based on their study requirements should provide researchers with a good first selection of wearables. Afterwards, they can narrow it down to the optimal wearable by further inspection of details, for example that on validity.

The two presented scenarios showed substantially different research goals, one of obtaining detailed information on the daily operation of physiological systems during a short measurement to predict future disease risk, the other to obtain insight into the temporal dynamics of physical activity, affect, and physiological arousal over a prolonged period within individuals. The suitability of a wearable for these typical short-term and long-term scenarios was added as a separate score to the SiA-WD to assist researchers in selecting the potential wearables for their research. Intermediate research scenarios exist that may require researchers to carefully formulate what is of importance.

### Limitations of the SiA-WD

A major limitation of the current version 1.0 of the SiA-WD is that it contains a modest selection of 54 devices, whereas over 172 devices were identified by our search. The 54 devices were selected for version 1.0 based on repeated use of a device in past stress research, input obtained from experts within the SiA consortium, and our aim to cover different types of devices (i.e., focusing on signals, wearable type, and consumer, research and clinical grade devices). While we succeeded in building a structure suitable for the wide variety of devices on the market, this first version may suffer from selection bias. The SiA-WD version 1.0 only includes wearables measuring autonomic nervous system reactivity, and no wearables capturing central nervous system activity. The gradual expansion of the database in the upcoming renewal cycles will at some point add these devices too and gradually reduce other selection biases. The continued renewal also ensures that we can pick up new promising devices that move from prototype to product in this rapidly moving field of technology and highlight the need for their subsequent validation, promoting the use of new technology. For example, we are especially awaiting to include in the database noninvasive wearables assessing continuous blood cortisol levels which may interest many stress and health researchers (Parlak et al., 2018).

It is not untypical in ambulatory research to extract the raw data recorded by the wearable and subsequently conduct preprocessing and parameter extraction in an open-access third-party platform such as Python or R. Upon the extraction of raw data, one might either use a more generalist package such as *neurokit2* that can be implemented to data from many wearables (Makowski et al., 2021) or utilize a more streamlined toolkit that was built for particular wearables, like the Wearables International repository. To facilitate this, we report in the SiA-WD whether the wearables enable raw data extraction as specified by the manufacturer.

Another limitation is that the SiA-WD is now maintained by a relatively modest-sized team. Not all candidate devices can be annotated for the database at once, as substantial work is involved per device (the time investment ranges from 0.5 to 5 workdays). Along with the rapid expansion of the total pool of wearables, this means that the curators may not keep up and will need to prioritize which devices to annotate first. Basic rules for this prioritization are that a device, listed in no order of importance, (1) has a novel or attractive feature (e.g., new way to monitor a widely used physiological parameter, large extension of battery life), (2) combines multiple physiological signals relevant to stress research (e.g., a device measures continuous ECG, BP, and EDA), (3) has the potential to substantially decrease participant burden (e.g., a ring rather than a patch), or (4) has been repeatedly used for peer-reviewed stress research and is still in-market. Also, the SiA-WD will maintain a clear focus on noninvasive wearables, therefore excluding ingestibles, implants, and domotic sensors. However, just like through the use of ASReview, we will stay open to implementing technologies that could benefit the maintenance of the database. For example, with the continuous improvements in the field of artificial intelligence (AI), large language models (LLMs) could potentially be implemented in the future to synthesize the RVU information. Nonetheless, with the variety of tasks involved in populating the SiA-WD, we see the human-in-the-loop principle as central to the decision-making process. We believe that in this way, the sustainability of the database will not be dependent on a given technology, and as a result, SiA-WD will be more responsive to change, including adoption of new and beneficial tools.

## Conclusion

The Stress in Action Wearables Database (SiA-WD) is a comprehensive and well-sustained database of physiological wearable devices that have application potential in behavioral research, in particular stress research. It provides a large amount of information that a researcher would look for such as the general device information, recorded signals, technical specifications and data access, combined with a systematic reliability, validity, and usability review of the available literature on a device. The SiA-WD will be iteratively expanded and the information, including that for devices already existing in the database, updated for a period of at least 10 years. A user-friendly tool will enable researchers to conveniently select the most suitable wearable for their study. The wearable database will continue to be moderated by a team of SiA researchers, but a future goal is to allow a broader group of researchers to actively contribute to this effort. They could propose devices to be added, point to additional identified reliability, validity, and usability studies, and suggest other points of information to be added to the database based on their user experience.

## Data Availability

Upon request, the individual scores of the curators along with the code used to calculate the inter-rater reliability can be provided. The database is openly accessible at https://osf.io/umgvp/.

## Declarations

### Open practices statement

The work and materials presented in the current paper were not preregistered.

All data and materials, including the database, are openly available at https://osf.io/umgvp/.
